# Emergency surgery due to diaphragmatic hernia: case series and review

**DOI:** 10.1186/s13017-017-0134-5

**Published:** 2017-05-18

**Authors:** Mario Testini, Antonia Girardi, Roberta Maria Isernia, Angela De Palma, Giovanni Catalano, Angela Pezzolla, Angela Gurrado

**Affiliations:** 1 Unit of Endocrine, Digestive, and Emergency Surgery, Department of Biomedical Sciences and Human Oncology, University Medical School “Aldo Moro” of Bari, Bari, Italy; 2 Department of Thoracic Surgery, University of Bari, Bari, Italy; 3 Unit of Laparoscopic Surgery, Department of Emergency and Organ Transplantation, University Medical School “A. Moro” of Bari, Bari, Italy

**Keywords:** Congenital diaphragmatic hernia, Diaphragmatic rupture, Mesh, Emergency surgery, Laparotomy, Thoracotomy

## Abstract

**Background:**

Congenital diaphragmatic hernia (CDH) is a congenital abnormality, rare in adults with a frequency of 0.17–6%. Diaphragmatic rupture is an infrequent consequence of trauma, occurring in about 5% of severe closed thoraco-abdominal injuries. Clinical presentation ranges from asymptomatic cases to serious respiratory or gastrointestinal symptoms. Diagnosis depends on anamnesis, clinical signs and radiological investigations.

**Methods:**

From May 2013 to June 2016, six cases (four females, two males; mean age 58 years) of diaphragmatic hernia were admitted to our Academic Department of General Surgery with respiratory and abdominal symptoms. Chest X-ray, barium studies and CT scan were performed.

**Results:**

Case 1 presented left diaphragmatic hernia containing transverse and descending colon. Case 2 showed left CDH which allowed passage of stomach, spleen and colon. Case 3 and 6 showed stomach in left hemithorax. Case 4 presented left diaphragmatic hernia which allowed passage of the spleen, left lobe of liver and transverse colon. Case 5 had stomach and spleen herniated into the chest. Emergency surgery was always performed. The hernia contents were reduced and defect was closed with primary repair or mesh. In all cases, post-operative courses were uneventful.

**Conclusion:**

Overlapping abdominal and respiratory symptoms lead to diagnosis of diaphragmatic hernia, in patients with or without an history of trauma. Chest X-ray, CT scan and barium studies should be done to evaluate diaphragmatic defect, size, location and contents. Emergency surgical approach is mandatory reducing morbidity and mortality.

## Background

Congenital diaphragmatic hernia (CDH) is an abnormality found in 1/2500 newborns, with a survival rate of 67% [1]. A primary characterization of CDH is that the diaphragm fails to form properly during embryogenesis. This incomplete formation of the diaphragm allows abdominal contents to herniate into the chest creating a mass-like effect that impedes lung development. Clinical presentation ranges from asymptomatic cases to serious respiratory or gastrointestinal symptoms, and sometimes haemodynamic instability. The broad spectrum of severity in patients with CDH is dependent on the degree of pulmonary hypoplasia and pulmonary hypertension. Posterolateral hernias (*Bochdalek* hernias) are the most common hernia type (>80%) with the majority occurring on the left side (85%), less frequently on the right side (13%) or bilateral (2%) [2].

Diaphragmatic rupture (DR) is an infrequent complication of trauma that occurs during 5% of trauma, including vehicle accidents [3–5]. Diagnosis is usually delayed; patients may be asymptomatic for years after trauma, until complications occur. Traumatic rupture of the diaphragm is considered an indication for surgical repair, especially in symptomatic patients [6].

However, there is no consensus on the absolute indications to surgery and about the timing. The onset of complications carries highest mortality and morbidity rates; therefore, it makes emergency surgery mandatory. During the past decades, primary suture repair or covering the defect with a synthetic mesh has been the standard procedures. More recently, biologic meshes have been thought to be effective in closing the diaphragmatic defect, inducing limited inflammatory response and minimizing adhesion formation [7]. Laparotomy or thoracotomy are the traditional treatments for patients with DR. Moreover, laparoscopic approaches for repair of hernias have recently gained in popularity [8]. Robotic approach is not yet described as effective approach in emergency, and it is reported in literature in only one case [9] in elective surgery.

This paper includes the surgical experience of congenital or traumatic diaphragmatic hernia of a surgical unit in emergency setting and reports the literature.

## Methods

Six cases of diaphragmatic hernia were observed in emergency at our Academic Department, with respiratory and abdominal symptoms. No breath sounds were detected in the left chest area, but bowel sounds were audible. Emergency surgery was performed in all cases. The hernia contents were reduced, and the defect was closed with primary repair or mesh.

**Fig. 1:**
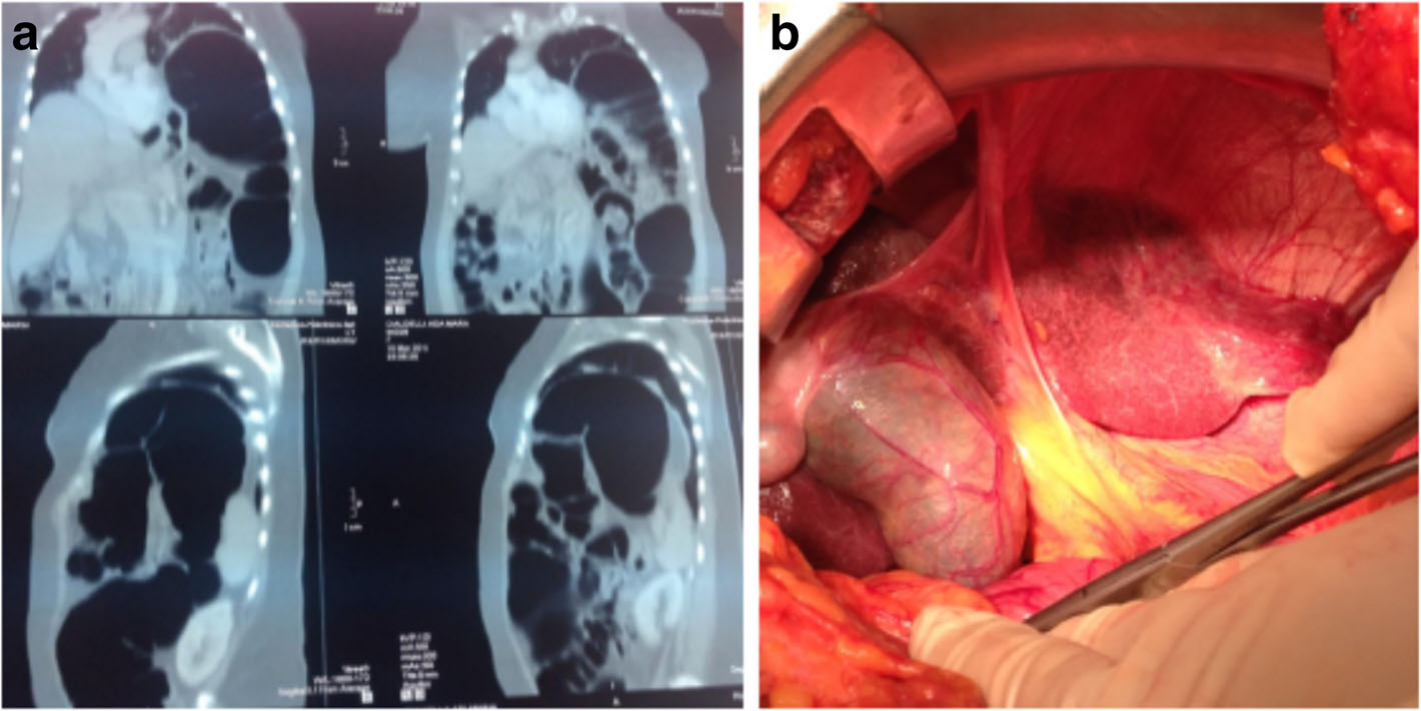
**a** CT scan shows collapse of the lung and the mediastinal shift towards to the *right side*. The *left* diaphragmatic hernia contained the transverse and descending colon. **b** Intraoperative evidence: diaphragmatic defect allows migration of viscera

Case 1: A 63-year-old woman was admitted with complaints of bowel obstruction and dyspnoea. Anamnesis revealed chronic abdominal pain, mental retardation and strabismus. In the physical examination, no breath sounds were detected in the left chest area; however, bowel sounds were audible. Chest X-ray and barium enema showed the transverse colon displaced into the left hemithorax above the splenic flexure. Computed tomography suggested collapse of the lung and the mediastinal shift towards the right. The left diaphragmatic hernia contained the transverse and descending colon (Fig. 1a). Emergency laparotomy was performed, and a left diaphragm agenesis, mega colon (diameter 10 cm) and left liver agenesis were found. An intra-operative bronchoscopy revealed hypoplasia of the left lung (Fig. 1b). A subtotal colectomy with ileo-rectal anastomosis was performed, and primary repair of diaphragm was done. The post-operative course was uneventful, and the patient was discharged on the 15th post-operative day. The research of abnormalities of the karyotype, phenotype and genetic pattern was negative for all the known congenital syndromes.

**Fig. 2:**
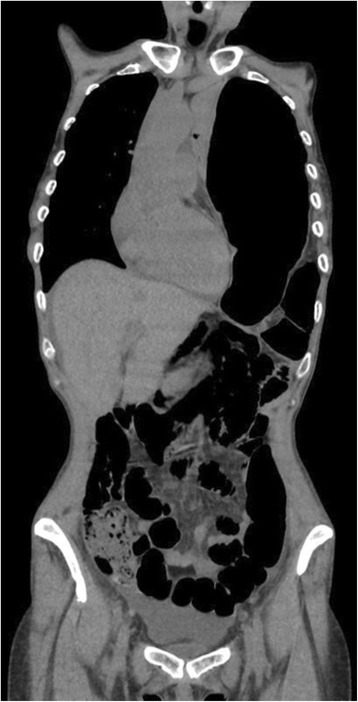
CT scan shows in *left side*, large diaphragmatic defect which allows passage of the stomach, spleen and colon (referred to as *Bochdalek* hernias) and complete collapse of left lung

Case 2: A 50-year-old woman was admitted with complaints of dyspnoea, chest and abdominal pain. No breath sounds were detected in the left chest area. There was no history of trauma. Chest X-ray revealed mediastinal shift towards the right and bowel gas in the left chest. CT scan showed large annular diaphragmatic defect which allowed passage of the stomach, spleen and colon (Fig. 2). An emergency combined chest-abdominal approach was performed, and contents were reduced repairing the defect with *Mersilene* mesh^®^. Thoracotomy approach was used to release the thoracic dense adhesion between the chest and the abdominal contents. Before placing the mesh, the anaesthesiologist increased the tidal volume to expand the collapsed left lower lobe of the lung and a chest drain was placed in the left pleural space. Immediate post-operative chest X-ray showed expansion of the left lung with minimal pleural effusion. Post-operative course was uneventful, and post-operative stay was 13 days.

**Fig. 3:**
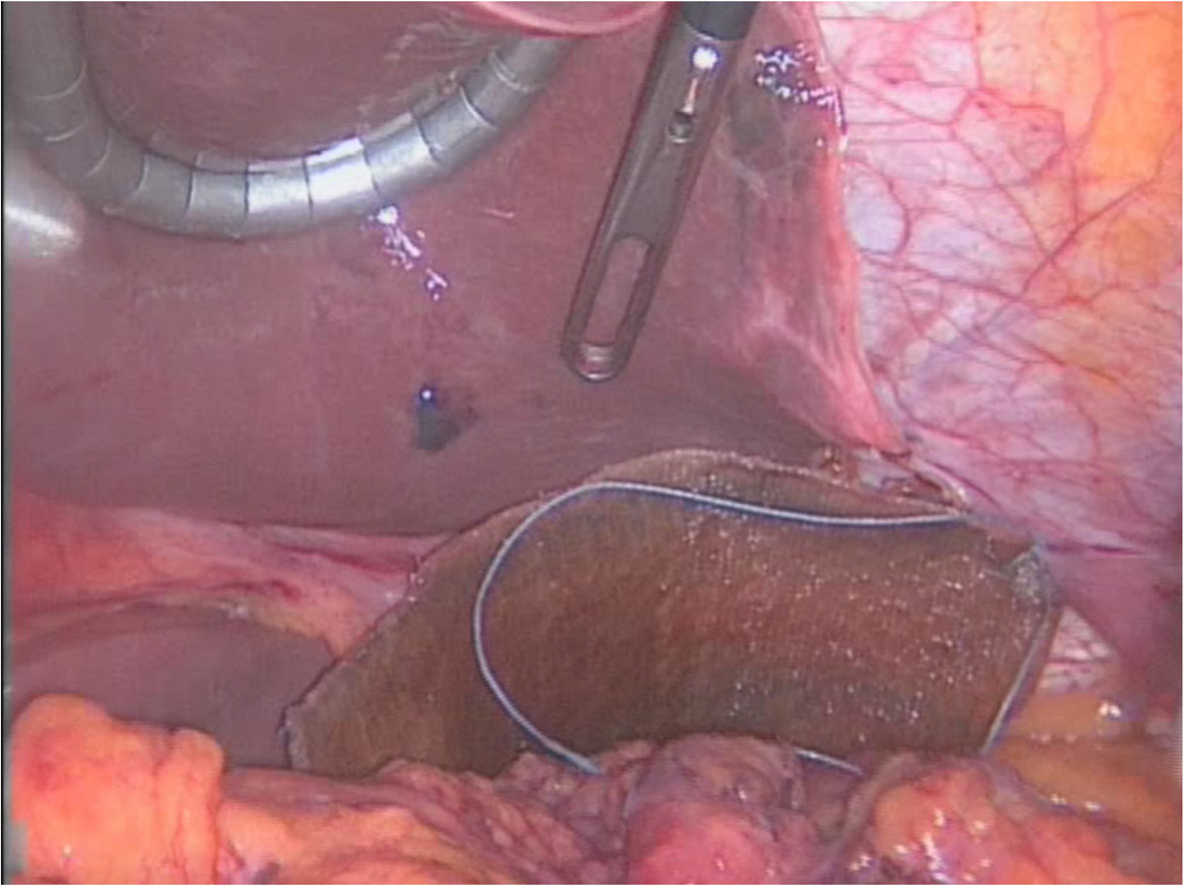
Laparoscopic image during correction of defect with synthetic mesh

Case 3: A 73-year-old woman arrived with complaint of breathlessness and dysphagia. No history of trauma was evident in anamnesis. Her current medical history included hypertension and hypothyroidism. Chest X-ray and barium studies demonstrated the presence of stomach in left hemithorax. CT scan revealed the presence of large diaphragmatic hernia which allowed the stomach to herniate into the chest. Emergency laparoscopy was performed; hernia contents were reduced; and a repair of the defect with *Proceed* mesh^®^ was done (Fig. 3). The post-operative course was uneventful, and patient was discharged 7 days after surgery.

**Fig. 4:**
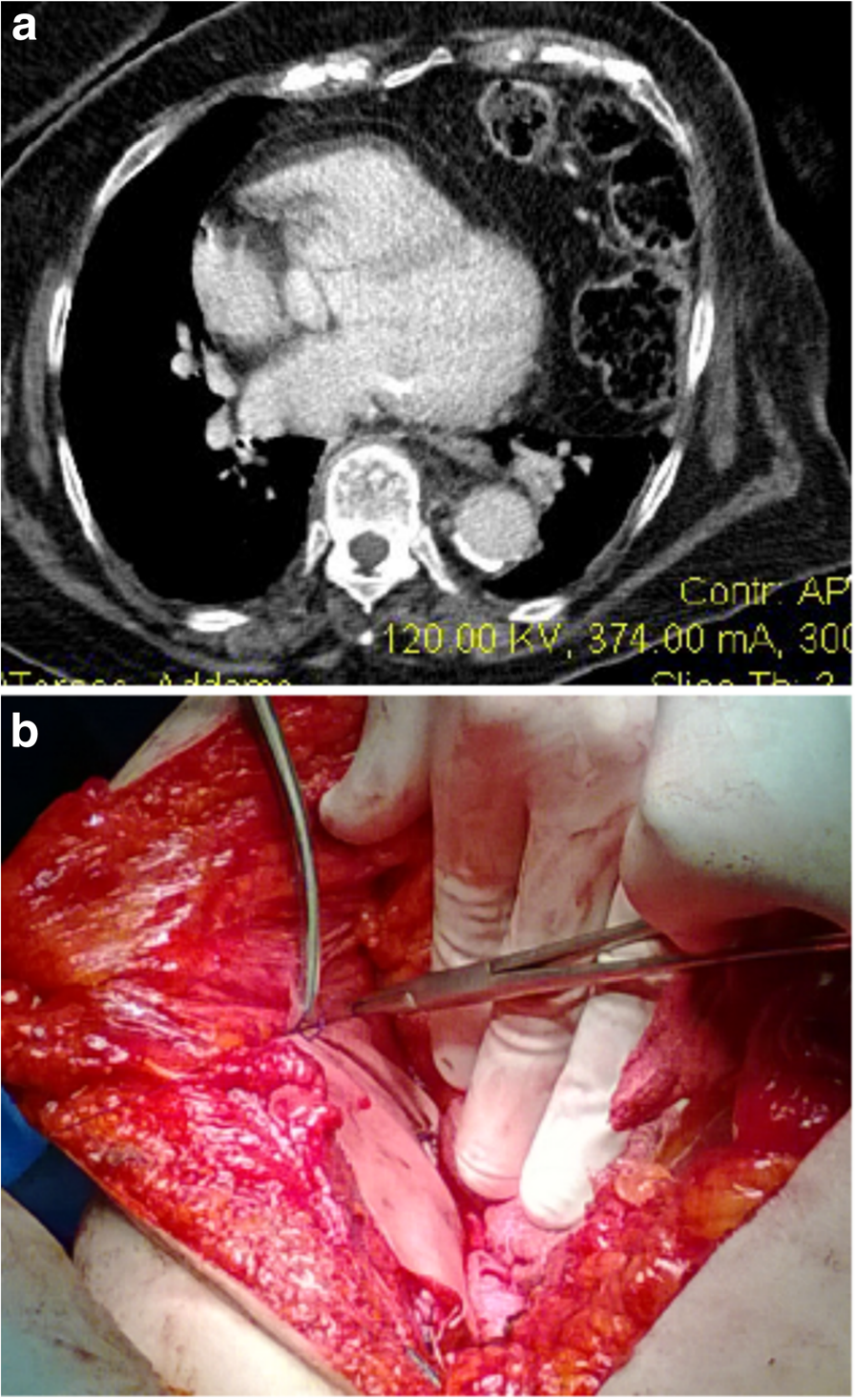
**a** CT scan suggests *left* diaphragmatic hernia which allowed migration of colon in *left* chest. **b** Intraoperatively, biological mesh repairing defect

Case 4: A 63-year-old woman was admitted with complaints of breathlessness for 2 days, which was gradually progressive and associated with left-sided chest pain and a dry cough. There was a history of a vehicle accident 6 years ago. The initial chest radiograph revealed an elevated left hemi diaphragm with presence of a colon gas shadow in the lower half of the hemithorax. CT scan suggested left diaphragmatic hernia which allowed passage of the spleen, left lobe of liver and transverse colon (Fig. 4a). Surgery was performed in emergency, reducing contents and repairing the defect with biological mesh (Fig. 4b; *Tutomesh, bovine pericardium mesh* ^*®*^). The patient was discharged on the 10^th^ post-operative day, without complications.

**Fig. 5:**
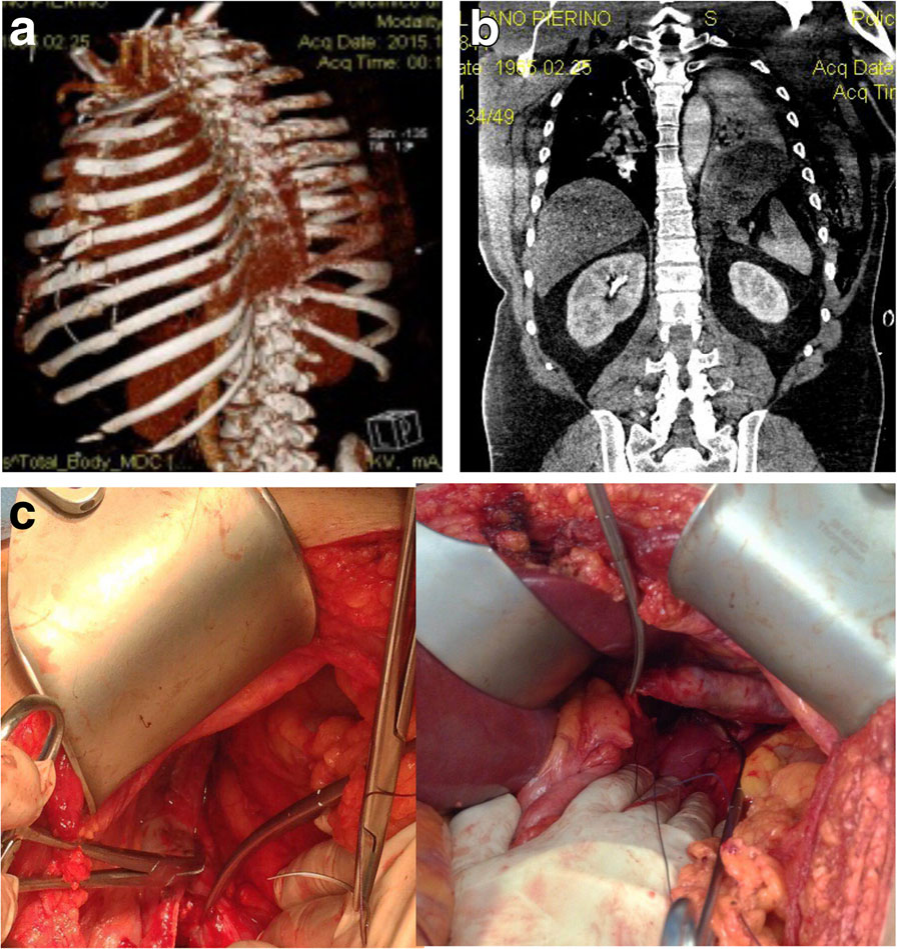
**a** 3D-CT scan shows rib fractures. **b** CT scan shows stomach and spleen in the *left* hemi-thorax, complete disruption of all muscular layers. **c** Intraoperatively, repair of traumatic defect in the *left* diaphragm

Case 5: A 50-year-old man was involved in a work accident. He was managed in accordance with *Advanced Trauma Life Support* protocol. He arrived in the emergency room with decreased breath sounds on the left side, dyspnoea, fever, left hypochondrium hematoma, subcutaneous emphysema, and chest and abdominal pain. His current medical history included obesity and treated hypertension. Initial chest radiography and barium studies demonstrated stomach in the left hemithorax. CT scan revealed stomach and spleen in left hemithorax, consistent with a traumatic diaphragmatic rupture with complete disruption of all muscular layers, collar sign and multiple rib fractures, fractured left humerus and scapula (Fig. 5a, b). At exploratory laparotomy, traumatic defect in the left diaphragm was found, with stomach and spleen in the left thorax (Fig. 5c). The hernia contents were reduced and the defect was closed with biologic mesh *(Tutomesh bovine pericardium mesh* ^*®*^). Post-operatively, the patient was placed in an intensive care unit. He was transferred from the ICU on the 8th post-operative day and discharged on the 20th day.

**Fig. 6:**
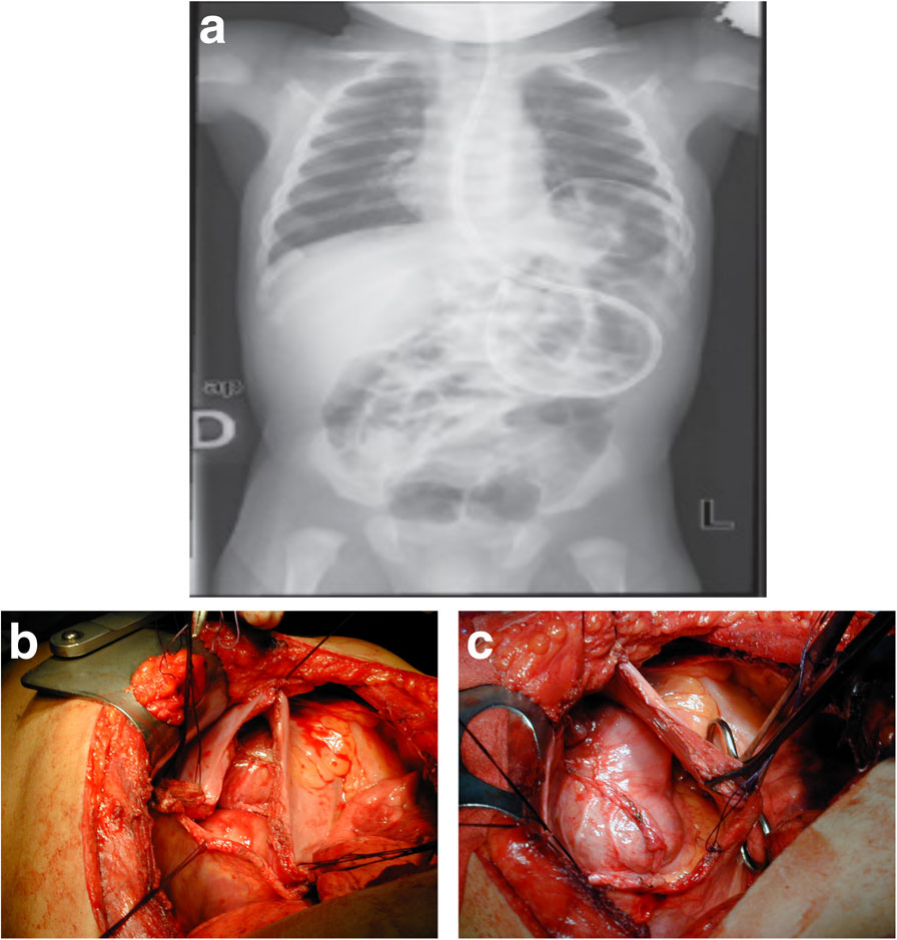
**a** X-ray shows herniated stomach into the chest. **b** Thoracotomy shows large diaphragmatic defect. **c** Repair of defect

Case 6 [10]: A 51-year-old man, referred to a history of 5 months of dyspnea, abdominal pain, nausea and vomiting. These symptoms had increased in severity during the previous 2 weeks. Anamnesis revealed left splenopancreatectomy 4 years earlier for non-*Hodgkin’s* lymphoma. The physical examination revealed a moderate peritoneal effusion without a peritoneal reaction. The introduction of a nasogastric tube remarkably improved symptoms. The chest X-ray showed a large fluid level beneath an apparently raised left hemi diaphragm (Fig. 6a) hypothesizing a left hemi diaphragmatic rupture with gastric herniation; diagnosis was confirmed by barium studies and a thoracic-abdominal computed tomography. An emergency left thoracotomy was performed, revealing a volvulus of the stomach, with some intestinal loops. Part of the transverse colon was incarcerated herniating through the torn diaphragm. The hernia was localized into the posterior side of the left hemi diaphragm with a diameter of 12 cm. During surgery, dense adhesions between the herniated organs and the left pleura-lung, as well as a marked reduction in left lung volume and an inflammatory mass in the greater omentum adherent to the diaphragm, were found. Thus, a reduction of the volvulus, an adhesiolysis and a resection of the mass were performed. Finally, a direct suture of the left diaphragmatic defect was employed (Fig. 6b, c). The patient had an uneventful recovery and histology showed Hodgki’s lymphoma.

### Review of the literature

**Fig. 7:**
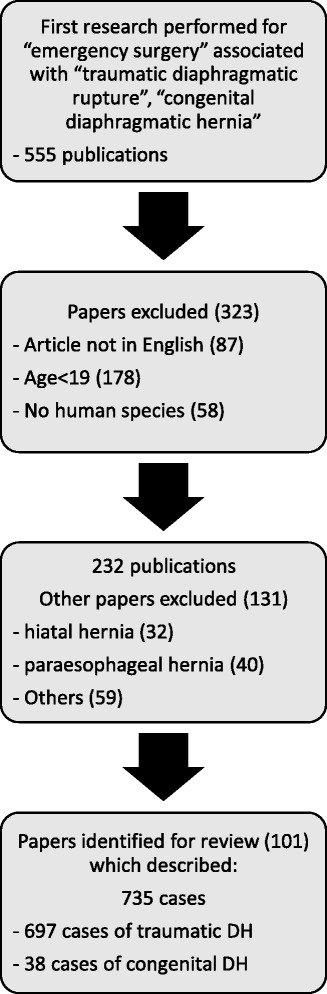
Flow chart of the literature selection process

A systematic review was performed by consulting PubMed/MEDLINE from 1983 to 2017 using the terms “emergency surgery”, associated with “traumatic diaphragmatic rupture”, and “congenital diaphragmatic hernia”. The search returned 555 papers (Fig. 7). Three hundred twenty-three publications were excluded because these articles were not written in English (*N* = 87), presented cases in childhood (<19 years old; *N* = 178) or were not interesting human species (*N* = 58); 32 papers were excluded because regarded hiatal hernia, 40 paraesophageal hernia and 59 elective setting. Consequently, the full texts of 101 articles were assessed for eligibility: the ethiopathogenesis was traumatic in 697 patients and congenital in 38 (Table 1).

**Table 1:** Review of literature showing demographics data, diagnosis and treatment

Authors, references	Number of patient, sex, age (years)	Aetiology	Diagnosis	Treatment	Type of hernia	Herniated organs
Lu J et al. Medicine 2016 [41]	1, M, 51	Traffic accident	Barium enema CT scan	Splenectomy	Left hemi diaphragm	Splenic flexure of the colon
	1, M, 45	Traffic accident	Chest X-ray, gastrografin contrast	Splenectomy	Left hemi diaphragm	Stomach and small bowel
	1, M, 47	Traffic accident	Chest X-ray, gastrografin contrast	Splenectomy	Left hemi diaphragm	Stomach and omentum
	1, M, 30	Traffic accident	Chest X-ray, Gastrografin contrast	Nonoperative treatment	Left hemi diaphragm	Stomach and omentum
	1, M, 33	Traffic accident	Chest X-ray, gastrografin contrast	Nonoperative treatment	Left hemi diaphragm	Stomach and omentum
	1, M, 29	Penetrating injury	Chest X-ray, gastrografin contrast	Nonoperative treatment	Left hemi diaphragm	Stomach and omentum
Manabu Harada, Int J Surg Case Rep. 2016 [42]	1, M, 78	Bochdalek hernia	Chest radiography and computed tomography	Laparoscopic Primary closure	Left hemi diaphragm	Omentum, transverse colon, and small intestine
De la Cour CD; Ugeskr Laeger. 2016 [43]	1, F, 27	Partum	Chest radiography and computed tomography	Primary closure	Left hemi diaphragm	
Razi K; J Surg Case Rep. 2016 [44]	1, F, 83	Morgagni hernia	Chest radiography and computed tomography	Mesh closure	Left hemi diaphragm	Transverse colon, greater curvature of the stomach and a partial gastric volvulus
Manson HJ Ann R Coll Surg Engl. 2016 [45]	1, F, 30	Bochdalek hernia	Chest radiography and computed tomography	Total gastrectomy with primary Roux-en-Y reconstruction, splenectomy and insertion of a feeding jejunostomy	Left hemi diaphragm	Gangrenous stomach and spleen, cardiac arrest
Massloom HS; N Am J Med Sci. 2016. [46]	1, M, 50	Bochdalek hernia	Computed tomography	Laparotomy and thoracotomy for repairing of defect	Left hemi diaphragm	Bowel
Kumar, J Surg Case Rep. 2016 [47]	1, M, 80	Morgagni hernia	Computed tomography	Laparotomy primary suture	Left hemi diaphragm	Gastric outlet obstruction
Manipadam JMJ Clin Diagn Res. 2016 [48]	1, M, 23	Bochdalek hernia	Chest X-ray	Laparotomy, sleeve resection of the gangrenous portion of the stomach	Left hemi diaphragm	Organoaxial volvulus of the stomach
Harada M, Int J Surg Case Rep. 2016 [49]	1, M, 78	Bochdalek hernia	Chest radiography and computed tomography	laparoscopic repair with primary closure	Left hemi diaphragm	Omentum, transverse colon, and small intestine
Siow SL; J Med Case Rep. 2016 [50]	1, M, 32	Traffic accident	Computed tomographic scan	Laparoscopic surgery with synthetic mesh repair	Left hemi diaphragm	
A.L. Andreev JSLS 2010 [51]	1, M, 40	Traffic accident 12 years earlier	CT scan	Laparoscopic primary suture	Left hemi diaphragm	Large intestine and greater omentum and acute colon obstruction
	1, M, 46	Surgery for a stab wound to the chest with injury to the heart 5 months before	Chest X-ray	Laparoscopic primary suture	Left hemi diaphragm	Transverse colonic segment
Bhatt NR, Trauma Mon. 2016 [52]	1, M, 23	Multitrauma 2 y before	Chest X-ray and CT scan	Laparotomy, adhesiolisis and primary repair	Left hemi diaphragm	Small bowel, omentum and large bowel obstruction
Abdullah M, Stonelake P BMJ case rep 2016 [53]	1, F 65	Trauma	Chest X-ray, CT scan	Emergency operation, laparotomy	Left hemi diaphragm	Perforated colon
Razi K; Journal of Surgical Case Reports, 2016 [54]	1, F, 83	Diaphragmatic Morgagni Hernia	Chest X-ray and CT scan	Laparoscopic repair with a composite mesh with an absorbable tic fixation on the diaphragm	Left hemi diaphragm	Transverse colon, the greater curvature of the stomach with a partial gastric volvulus
A Wigley J Ann R Coll Surg Engl 2014 [55]	1, F, 72	Traffic accident				
Atef Mejri Medicine 2015 [56]	1, M, 56	Bochdalek hernia	Chest X-ray, barium studies and CT scan	Primary repair Laparoscopy was converted laparotomy	Left hemi diaphragm	Gastric volvulus
Mahmut Tokur Ulus Travma Acil Cerrahi Derg, July 2015 [57]	1, F, 27	Congenital DH	Chest X-ray, CT scan	Thoracotomy, primary repair	Left hemi diaphragm	Gastro thorax
Topuz Mustafa Ulus Travma Acil Cerrahi Derg. 2014 [58]	1, F, 55	Traffic accident	Chest X-ray, CT scan	Laparotomy primary repair	Right hemi diaphragm	Liver causing mechanic compression on ventricle
Moussa G Ann R Coll Surg Engl. 2014 [17]	1, F, 65	Previous history of pericardial window fenestration and sarcoidosis	Chest X-ray, CT scan	Laparoscopy, mesh repair	Right hemi diaphragm	Left lobe of liver, stomach and colon
Nakamura T, Ulus Travma Acil Cerrahi Derg. 2014 [18]	1, M, 81	History of HCC treated with Radiofrequency ablation	Chest US, CT scan	Laparotomy, primary hernia repair, small bowel resection	Right hemi diaphragm	Liver, incarcerated small bowel
Haratake Naoki Surgery today 2015 [59]	1, F, 50		CT scan	Laparotomy, primary hernia repair	Right hemi diaphragm	Heterotopic endometriosis in a patient with Chilaiditi syndrome
Gali BM, Niger J Med. 2014 [60]	1, M, 28	Penetrating injury years before	CT scan	Laparotomy, primary repair	Left hemi diaphragm	Bowel
Michael Joseph Newman, BMJ Case Rep 2014 [61]	1, M, 25	Bochdalek hernia	Chest X-ray, CT scan	Laparotomy, primary repair, gastric resection	Left hemi diaphragm	Stomach and bowel
Tyagi Sam, Ann Thorac Surg. 2014 [62]	1, M, 36	Morgagni hernia	Chest X-ray, CT scan	Laparoscopy Gore-Tex fixed with a spiral tacker	Left hemi diaphragm	Omentum and transverse colon
Kurniawan N, Acta Chir Belg. 2013 [32]	1, M, 17	Bochdalek hernia	Chest X-ray, CT scan	Laparoscopy primary sutture	Left hemidiaphragm	Stomach, spleen colon
Ota H Ann Thorac Cardiovasc Surg. 2014 [63]	1, M, 62	Fall accident	ECO FAST, Chest X-ray, CT scan	Video assisted mini thoracotomy Primary suture	Right hemi diaphragm	Hemothorax
G, et al. BMJ Case Rep 2013 [64]	1, M, 60	Fall	Chest X-ray, CT scan	Laparoscopy and laparotomy	Left diaphragm	Stomach, bowel and spleen
Sonthalia N, J Emerg Med. 2013 [65]	1, F, 78	Morgagni hernia	Chest X-ray, CT scan, barium studies	Thoracotomy	Left diaphragm	Gastric volvulus
Nayak HK BMJ Case Rep. 2012 [66]	1, M, 50	Blunt trauma	EGDS, barium studies, CT SCAN	Laparoscopic repair	Left hemi diaphragm	Gastric volvulus and duodenum
Vernadakis S, Transplant Proc. 2012 [67]	1, F, 46	Liver donor	Chest X-ray, CT scan, barium studies	Laparotomy	Right diaphragm	Bowel
Ngai I, BMJ Case Rep. 2012 [68]	1, F, 31	Pregnancy	MRI	Nasogastric tube	Left hemi diaphragm	Spleen, bowel, stomach and pancreas
Elangovan A J Emerg Med. 2013 [69]	1, M, 30	Accident	Chest X-ray and CT scan	Laparoscopy	Left hemi diaphragm	Stomach
Kuppusamy A, Ulus Trauma Acil Cherrai Derg 2012 [70]	1, M, 28	Trauma	CT scan	Thoracotomy	Right hemi diaphragm	Liver
Ismail Okan, Ulus Travma Acil Cerrahi Derg. 2011 [71]	10 cases, 44,3 y	Trauma	CT scan	7 laparotomy 1 thoracic-abdominal approach 2 thoracic	9 left side	
Ioannis Baloyiannis General Thoracic and Cardiovascular Surgery 2011 [72]	1, M, 56	Trauma		Laparotomy		
Vassileva CM Ann Thorac Cardiovasc Surg. 2012 [73]	1, F, 25	Morgagni hernia	Chest X-ray, CT scan	Laparoscopic repair	Right hemi diaphragm	Omentum
Agrafiotis AC Acta Chir Belg. 2011 [74]	1, F, 52	Bochdalek hernia	Chest X-ray, CT scan	Laparoscopic approach, and mini laparotomy prosthetic polypropylene mesh	Left hemi diaphragm	Small bowel loops and the right colon
Tan K K, Singapore Med J 2009 [75]	14, median age 38 y	Trauma	Chest X Ray, CT Scan, RMN	Laparotomy, thoracotomy or VATS Primary repair (85.7%) patients or patch repair	five (35.7%) right-sided and nine (64.3%) left-sided diaphragmatic ruptures	
Akhtar K, Br J Hosp Med (Lond). 2009 [76]	1, M, 27	Bochdalek hernia	Chest X Ray, Upper gastrointestinal endoscopy, CT scan	Laparoscopy Goretex dual mesh	Left hemi diaphragm	Small bowel, ascending and transverse colon, and spleen
Ozpolat B, Ulus Travma Acil Cerrahi Derg. Nov; 2009 [77]	1, M, 52	Tube thoracostomy at the seventh left intercostal	Chest X-ray, MRI	Left standard thoracotomy, primary suture	Left hemi diaphragm	Omentum
Altinkaya N Hernia. 2010 [78]	12 patients mean age of 60 years, ten were female.	Morgagni hernia	CT scan	Six patients had surgery. 1 emergency surgery for hernia, 2 laparoscopic hernia repair, 3 trans-abdominal repair and 1 transthoracic repair	Right hemi diaphragm	Omentum and colon
Syed Murfad Peer, Int J Surg. 2009 [79]	2496 patients25 (86%) males4 (14%) females mean age 33.6 y	Trauma	Chest X-ray diagnostic in 20 (69%) patients CT scan in 4 (14%) patients. Intra-operative diagnosis of rupture diaphragm was made in 5 (17%) patients.	29 (1.1%) underwent to surgery 20 thoracotomy (69%) 8 laparotomy (27.5%) 1 Thoracoabdominal approach (3.5%)	Right defect: 6 left defect:23	
Sung HY J Korean Med Sci. 2009 [80]	1, F, 49	Congenital hernia	Chest radiography	Thoracotomy	Left hemi diaphragm	Stomach, spleen, splenic flexure of the colon bowel loops
Ouazzani A Acta Chir Belg. 2009 [81]	1, M, 24	Trauma	Chest X-ray computed tomography	Laparoscopically, with mesh	Left diaphragm	Stomach
Kavanagh D Acta Chir Belg. 2008 [82]	1, M, 76	Bochdalek hernia	Chest radiograph and computed tomogram	Laparotomy, primary repair	Right diaphragm	Strangulation of a portion of transverse colon
Yeh-Huang Hung; J Chin Med Assoc. 2008 [83]	1, M, 74 1, F, 75	Bochdalek hernia Bochdalek hernias	Chest X-ray CT scan MRI	Laparotomy Transthoracic repair	Left diaphragm Right diaphragm	Intestinal obstruction Small and large bowels
Sano A Surg Today. 2008 [16]	1, F, 25	Diaphragm hernia during pregnancy	Chest radiograph and computed tomography	Emergency caesarean section sutures and a Gore-Tex sheet	Left diaphragm	Bowel loop
Gourgiotis S, Turkish Journal of Trauma & Emergency Surgery 2008 [84]	1, M, 25	Trauma	Chest X-ray CT scan	Laparoscopic primary repair	Left diaphragm	
Walchalk LR, J Emerg Med. 2010 [85]	1, F, 57	Trauma				
Mohammadhosseini B, J Coll Physicians Surg Pak. 2008 [86]	1, M	Bochdalek hernia				
Boyce S, Obes Surg. 2008 [87]		Diaphragmatic hernia post surgery	CT of the chest and abdomen	Laparotomy an repair of hernia	Left diaphragmatic hernia	Ischemic small bowel
Tsuboi K, Surg Today. 2008 [88]	1, M, 50	16 months after surgery	Computed tomography of the chest	Laparotomy	Left diaphragmatic hernia	Stomach had herniated into the thoracic cavity
Vogelaar Obes Surg. 2008 [89]	1, F, 37	Six months after gastric banding	Chest X-ray computed tomography scan	Laparotomy	Left diaphragm	Intra thoracic stomach distended, rotated, and perforated at the orifice of the hernia
Young-Shun Wu; Am J Emerg Med. 2008 [90]		History of left-sided upper abdominal blunt injury 2 months before	CT scan	Thoracotomy and primary repair	Left traumatic diaphragm rupture	
Igai H, Y Gen Thorac Cardiovasc Surg. 2007 [91]	1, M, 48	Trauma	Chest X-ray, CT scan		Right diaphragm rupture	Hepatothorax
Rifki Jai S Arch Gynecol Obstet. 2007 [92]	1, F, 27	32-week gestation no history of trauma	Chest X-ray CT scan	Emergency laparotomy	Left hemi diaphragm.	Stomach, transverse colon and greater omentum herniated in the left hemithorax
Rout S Hernia. 2007 [93]	1, F, 35	Bochdalek hernias	Chest X-ray CT scan	Emergency laparotomy defect was repaired using non-absorbable sutures	Right-sided Bochdalek hernia	Colon
Campbell AS Hernia. 2007 [94]	1, M, 85		Chest X-ray CT scan	Emergency laparotomy identified a massive diaphragmatic defect which was not amenable to primary closure. A colopexy procedure was performed	Left hemi diaphragm.	Diaphragmatic herniation of bowel
Testini M Surg Today. 2006 [10]	1, M, 51	Left splenopancreatectomy 4 years earlier	Chest X-ray, CT scan, MRI	Left thoracotomy	Left hemi diaphragm	Stomach
Luu TD, Ann Thorac Surg 2006 [95]	1, F, 34	33 weeks’ gestation	Chest roentgenogram, CT scan, barium study Esophagoscopy	the patient went into preterm labour and had a spontaneous vaginal delivery of a healthy new-born at 34 weeks’ gestation. left thoracotomy	Left hemi diaphragm	Necrotic stomach
Iso Y., Hernia 2006 [96]	1, F, 81	Morgagni’s hernia	Chest X-ray	The diaphragm defect was sutured first, and partial resection of the transverse colon	Right thorax	transverse colon
Eglinton T, ANZ J Surg. 2006 Jul [97]	3 cases	During third trimester of pregnancy	Chest X-ray	Laparotomy and thoracotomy in one case. Delivery was by Caesarean section at the time of emergency surgery		
Barbetakis N, World J Gastroenter ol. 2006 Apr 21 [98]	1, F, 31	Bochdalek hernias during pregnancy (23-week gestation)	Chest X-ray, chest ultrasound	Left thoraco- abdominal incision, segmental resection of the involved portion of large bowel. The diaphragmatic defect was repaired with interrupted sutures	Left hemi thorax	Strangulated Right and transverse colon, necrotic the greater omentum and stomach
Barret J, J Emerg Med. 2006 [99]	1,M, 50	Trauma	Electrocardiogram and CT scan		Left hemi thorax and pericardium	
Abboud B, J Med Liban. 2004 [100]	1 M	Trauma	Chest X-ray, exploratory laparotomy	Laparotomy, colectomy resection of ileum with anastomosis	left hemi thorax	Transverse colon and a proximal small bowel
Hsu YP, Hepatogastroenterology. 2005 [101]	78 patients	Trauma	Chest roentgenogram	Only 20% of elderly patients were operated on within 24 h of trauma, 87% of young patients		
P Ransom Emerg Med J 2005 [102]	1, M, 21	Trauma	Chest radiograph, ultrasound, oesophago-gastro- duodenoscopy	Thoracotomy	Left diaphragm	Stomach and a loop of colon had herniated through a 6 cm defect
Tiberio GA Acta Chir Belg. 2005 Feb [103]	33 p	Blunt (22 patients) or penetrating injury	Chest X-ray, CT scan	Laparotomy		
Barakat MJ, BMC Surg. 2005 [19]	1, F, 43	CDH in Marfan’s syndrome	Chest X-ray, CT scan	Laparoscopy	Right hemi diaphragm	Perforated gangrenous appendix
Gupta V Eur J Emerg Med. 2005 [104]	1, M, 43	Spontaneous rupture	CT scan		Left hemi diaphragm	
Kara E Ann Acad Med Singapore 2004 [105]	1, M, 28	Trauma	Chest X-ray, CT scan	Left thoracotomy	Left hemi diaphragm	Gastric fundus
Sirbu H Hernia. 2005 [106]	1, M, 67	Trauma	CT scan	Laparotomy and right thoracotomy	Delayed bilateral diaphragmatic ruptures	
Dalton AM Emerg Med J. 2004 [107]	1, M, 43	Bochdalek hernia	Chest radiograph	Thoracotomy	Left hemi thorax	Stomach, transverse colon, and spleen in to the chest.
Niwa T Respiration. 2003 [108]	1, F, 53	Bochdalek hernia	Chest X-ray	Thoracotomy	Left hemithorax	Stomach and greater omentum
Genc MR, Obstet Ginecol 2003 [109]	1, M, 29	Bochdalek hernia during pregnancy	Chest X-ray, CT scan	Antepartum repair	Left hemithorax	Bowel obruction
Sato M, Jpn J Thorac Cardiovasc Surg. 2002 [110]	1, M, 57	Traffic accident	Chest X-ray, CT scan, MRI	Toracoscopy	Right hemidiaphragm	Liver
Guven H, Acta CHir Belg 2002 [111]	2 cases	Morgagni hernia				Bowel perforation Upper gastrointestinal bleeding
Kanazawa A, Surg Today 2002 [112]	1 F 63 y	Bochdalek hernia	Chest X-ray, CT scan,	Thoraco-Laparotomy Primary suture	Right hemidiaphragm	Colon and right kidney
Fisichella PM, Ann Ital CHIR 2001 [113]	1 F 55 y	Bochdalek hernia	Computed tomography	Thoracotomy and laparotomy	Right hemidiaphragm	Liver intestinal maloration
Bergeron E, J Trauma 2002 [15]	160 cases	Trauma				
Carreno G, Surg Endosc 2001 [114]	1, M, 52	Bochdalek hernia	CT scan	Laparoscopic approach	Left hemi thorax	Colon and volvulated stomach
Prieto Nieto I, Acta chir Belg 2001 [115]	1, M, 36	8 months after trauma	CT scan	Laparotomy, repair of defect, gastric perforation were closed	Left hemi diaphragm	Gastric incarceration and perforation
Nursal TZ, Hernia 2001 [116]	26 cases	Trauma	Chest X-ray, CT scan,	92% Primary repair	92% left hemi diaphragm	31.8% stomach 27,2 colon
Bujanda L, J Clin Gastroenterol 2001 [117]	1	Bochdalek hernia			Left hemi diaphragm	Gastric volvulus
Pross M, J Laparoendosc Adv Surg Tech A 2000 [118]	1, M, 20	Trauma	Diagnostic laparoscopy	Laparoscopy primary repair	Left hemi diaphragm	Stomach
Saito Y, Surg Today 2000 [119]	1, M, 51	3 years after pleuropneumectomy for mesotelioma		Laparotomy	Left hemi thorax	Ulcer of stomach, which was displaced into the thorax, had perforated aorta
De Waele JJ, J Accid Emerg Med 1999 [120]	1, M, 45	Trauma	Chest X-ray, Chest tube; ultrasound,	Laparotomy, resection of spleen	Left hemi thorax	Spleen completely disrupted
Colliver C, J Trauma 1997 [121]	1, M, 80	Trauma	Echocardiograph, ultrasonography		Left hemi diaphragm	Stomach Intrapericardial hernia
Zantut LF, Rev Paul Med 1993 [122]	1, M, 33	Trauma	Chest X-Ray, liver scintigraphy, CT scan, MRI, diagnostic laparoscopy	Laparoscopy	Bilateral diaphragmatic rupture	
Allen MS, J Thorac Cardiovasc Surg 1993 [123]	147 cases 5 emergency setting		Chest X-ray CT scan		Left hemi diaphragm	Stomach
Girzadas DV Jr Ann Emerg Med. 1991 [124]	1, F, 71	Trauma	Chest radiograph		Pericardial sac	Omentum and transverse colon
Thomas S Jpn J Surg. 1991 [125]	2 cases	Bochdalek hernia	X-ray of the chest and contrast studies of the gastrointestinal tract	Laparotomy	Left hemi diaphragm	Intestinal obstruction
Bush CA, South Med J 1990 [126]	2 cases	Trauma	Chest X-ray, barium studies of the gastrointestinal tract, CT scans, ultrasonography, laparoscopy, and radionuclide scanning	Laparoscopy	Left hemi diaphragm	Intestinal obstruction
Feliciano DV, J Trauma 1988 [127]	16 cases	Penetrating trauma	Chest X-ray	Laparotomy		
Chidamdaram M Thorac Cardiovasc Surg. 1988 [128]	1, M, 32	Trauma	Chest X-ray	Thoracotomy	Left hemi diaphragm	Stomach
Symbas PN, Ann Thorac Surg 1986 [129]	194 cases	Trauma	Chest X-ray, barium studies exploratory laparotomy	Laparotomy Primary repair In a case Prolene mesh		
Saber WL, J Emerg Med 1986 [130]	111 cases 8 emergency surgery	Trauma	Chest X-ray		7 left 1 right hemi diaphragm	
Gardezi SA, J Pak Med Assoc 1986 [131]	2 cases 1, M, 43	Bochdalek hernia	Chest X-ray	Laparotomy	Left hemi diaphragm	Transverse colon and splenic flexure
	1 M 26 y	CDH	Chest X-ray	Laparotomy	Left hemi diaphragm	Greater curvature of stomach, a small part of jejunum, left part of trans­verse colon and greater omentum
Brown GL, Ann Thorac Surg 1985 [132]	41 cases	Trauma	Chest X-ray	23 laparotomy, 13 thoracotomy, 5 combined	29 Left, 14 Right-sided, hemi diaphragm.	
Clark DE, Surgery 1983 [133]	10 cases median age 40	Trauma	Chest X-ray		Left hemi diaphragm	

*M* male, *F* female *Y* years

### Pathogenetic mechanism

Diaphragmatic rupture with abdominal organ herniation was first described in 1541 by *Sennertus* [11]. Congenital diaphragmatic hernias are prenatally or during the neonatal period diagnosed. On the contrary, CDH in adulthood are exceedingly rare and can occur through an anterior parasternal *Morgagni* foramen or through a posterolateral, mainly left-sided, named as Bochdalek hernia, firstly described in 1848 [12]. The aetiology is still under study, but the disease is due to the failure of closure of the canal between the *septum transversum* and the oesophagus during the 8th week of gestation. Morgagni hernia is a rare disease caused by the defective development of the sternal attachments to the diaphragm. Traumatic diaphragmatic hernias are thought to be produced by a sudden increase in the pleuroperitoneal pressure gradient occurring at areas of potential weakness along embryological points of fusion [13].

DR usually result from blunt or penetrating injuries or iatrogenic causes and result in entry of an abdominal hollow viscus or the omentum into the pleural cavity, which may lead to incarceration and even strangulation with a fatal outcome. Traumatic diaphragmatic hernias are frequently caused by a penetrating injury (10–19%), sometimes by blunt thoracic-abdominal trauma (5%) [14, 15]. Moreover, some authors described rare and particularly cases of DR after surgery or pregnancy; that is Sano A. et al. reported a case of a pregnant woman in the 28th week of pregnancy, who was underwent to emergency caesarean section and repair of the diaphragm [16]; Moussa G. et al., described a right DR in a patient with previous history of window fenestration and sarcoidosis [17]; Nakamura T. et al., reported a case of right DR in patient with a history of hepatic carcinoma treated with radiofrequency ablation [18]. Furthermore, there was an association between Marfan’s syndrome and CDH as *Barakat* et al. reported [19].

### Site of rupture

CDH formation is found 80% on the left side [20]. Also, 88–95% of diaphragmatic ruptures occurred on the left side [21], especially, blunt trauma causes large diaphragmatic defects, commonly involving (>80%) the left posterolateral diaphragm [22]. The right haemidiaphragm is stronger than the left one because of the size of the liver which has a protective effect. For this reason, the side ruptures are very rare and associated with high mortality and morbidity rate [23].

The review of literature reported in this study confirmed the high frequency of left defect 80%, and only two cases of bilateral DR were reported.

### Presenting symptom and investigations

*Nayak* et al. described severe symptoms, in 46% of CDH cases with 32% of mortality due to visceral strangulation [24]. Moreover, the literature analysis shows a variable rate of delayed symptoms (5–45.5%) [25, 26]. Late-presenting CDH of left sided typically produces acute, obstructive, gastrointestinal symptoms, chronic dyspnea, chest pain, recurrent abdominal pain, postprandial fullness and vomiting, evolving to cardiorespiratory failure [27]. Indeed, right-sided CDH is usually associated with only respiratory issues because partial liver displacement may block the further herniation of hollow viscera [1]. Although the presence of bowel sounds within the chest and the absence of breath sounds are typical findings associated with a CDH, a misdiagnosis rate of 38% has been reported [28]. Obviously, in totally asymptomatic cases, diagnosis is very hard. On the contrary, when acute presentations occur because of the increasing of abdominal pressure and consequent rapid visceral displacement into the chest or due to rapid distension of previously herniated viscera, diagnosis is clear [29, 30]. Chest X-ray and barium studies are useful for determining which viscera have herniated into the thorax. The most common reported radiological finding of CDH is the opaqueness of the hemithorax usually associated with mediastinal shift to the contralateral side. Moreover, the position of the nasogastric tube in the chest cavity will provide an important indicator and prompt correct diagnosis. Computed tomography can be considered the *gold standard* technique for diagnosis, offering the unique opportunity to evaluate the presence, size and location of a diaphragmatic defect, as well as the contents of various types of diaphragmatic hernias [31] and showing sensitivity and specificity of 14–82% and 87%, respectively [32]. MRI is also useful, but usually it is not performable in emergency. However, it is usually employed in stable patients or where the CT scan is equivocal [33]. According with literature, in this reported experience, a definitive diagnosis was made with CT scan and barium studies.

Late-presenting CDH is considered as a benign condition but it can rapidly becomes a life-threatening disease [1, 27, 28, 31, 33]; consequently, an immediate surgical treatment is mandatory. Associated anomalies in late-presenting CDH patients, such as congenital heart disease, *Fryns* syndrome and trisomy 18, have been reported in 8.6–80% of cases [1, 2, 27, 28], significantly increasing the mortality rate. At this proposal, in case 1, even if there was a high suspicion of congenital syndrome, surprisingly it was not confirmed by genetic studies.

### Surgical treatment

Surgical repair typically involves primary or patch closure of the diaphragm through an open abdominal approach. When the diagnosis is delayed, due to suspicions of adhesions between viscera and chest, thoracotomy or combined thoracic-abdominal approach is preferred, as in the reported case 2. Some authors have reported success with thoracoscopic approach but vitiated by an increased incidence of hernia recurrence [34–36]. Furthermore, during thoracoscopy, an intraoperative pulmonary hypertension with subsequent hemodynamic instability could develop; moreover, the placement and management of a patch results in substantially longer operating times. For these reasons, thoracoscopic repair of CDH is preferred in the presence of small diaphragmatic defects and/or mild pulmonary hypertension [37]. Nowadays, the laparoscopic approach is safe and feasible for CDH and it could be an excellent option [37], as in case 3.

However, emergency surgery is the treatment of choice for diaphragmatic rupture. In delayed cases, thoracic approach is recommended to reduce viscera-pleural adhesions and to avoid intra-thoracic visceral perforation with catastrophic complications [38]. When the suspicion of intestinal obstruction is evident, an abdominal approach may also be required to control organs. Although the type of closure used for diaphragmatic hernias is still a matter of debate, it is generally accepted that most defects can be primarily closed with a non-absorbable suture [39]. Mesh repair usually is used when the defect is too large to be primarily closed and the use of tension free mesh is vital to the success of the procedures. Recently, biologic mesh has been introduced to replace the synthetic one because of its lower rate of hernia recurrence, higher resistance to infections and lower risk of displacement [7, 40]; however, limited evidence in literature yet exists about their superiority. Indeed, in our previous experience, biologic meshes have also been used in contaminated surgical fields with favourable results [40]. However, because of the rarity of this condition, clinicians should be encouraged to publish their experience with biologic meshes in diaphragmatic hernia repair [7].

## Conclusions

When a diaphragmatic hernia is diagnosed, surgery is the treatment of choice, above all in emergency setting. A multidisciplinary approach in dedicated centres is advisable.

## Abbreviations

CDH: Congenital diaphragmatic hernia
CT: Computed tomography
DR: Diaphragmatic rupture
MRI: Magnetic resonance imaging
