# Correction to: Emergency surgery due to diaphragmatic hernia: case series and review

**DOI:** 10.1186/s13017-019-0269-7

**Published:** 2019-10-17

**Authors:** Mario Testini, Antonia Girardi, Roberta Maria Isernia, Angela De Palma, Giovanni Catalano, Angela Pezzolla, Angela Gurrado

**Affiliations:** 1 Unit of Endocrine, Digestive, and Emergency Surgery, Department of Biomedical Sciences and Human Oncology, University Medical School “Aldo Moro” of Bari, Bari, Italy; 2 Department of Thoracic Surgery, University of Bari, Bari, Italy; 3 Unit of Laparoscopic Surgery, Department of Emergency and Organ Transplantation, University Medical School “A. Moro” of Bari, Bari, Italy

**Correction to: World J Emerg Surg**

**https://doi.org/10.1186/s13017-017-0134-5**

The original article [1] contains a minor typo in reference 47 (reference [2] in this Correction article); the correct reference notation can be shown in the respective reference within this article.
